# Radiolucent Lesions of the Jaws: An Attempted Demonstration of the Use of Co-Word Analysis to List Main Similar Pathologies

**DOI:** 10.3390/ijerph19041933

**Published:** 2022-02-09

**Authors:** Andy Wai Kan Yeung

**Affiliations:** Oral and Maxillofacial Radiology, Applied Oral Sciences and Community Dental Care, Faculty of Dentistry, University of Hong Kong, Hong Kong, China; ndyeung@hku.hk

**Keywords:** radiolucency, jaw pathology, differential diagnosis, ameloblastoma, osseous dysplasia

## Abstract

(1) Background: Many radiolucent jaw lesions exist, and they often show a radiographic resemblance, rendering diagnosis a challenging act. Closely related lesions should be frequently mentioned together in the academic literature, which might be helpful for junior practitioners in determining their differential diagnosis. The usefulness of bibliometric analysis in this respect has yet to be demonstrated. (2) Methods: This study evaluated academic publications on radiolucent jaw lesions, as indexed by the Web of Science Core Collection database. The mentions of radiolucent jaw lesions were extracted from the complete bibliographic records of the publications, and co-word analyses were conducted with the aid of VOSviewer. (3) Results: Based on 1897 papers, visualization maps were synthesized to evaluate co-occurrences of the radiolucent jaw lesions. Ameloblastoma was frequently mentioned together with odontogenic keratocyst, dentigerous cyst, and radicular cyst. Osseous dysplasia was co-mentioned with osteomyelitis, ossifying fibroma, odontoma, fibrous dysplasia, and apical periodontitis. (4) Conclusions: The co-word analysis, a form of bibliometric analysis, could demonstrate a relatedness of radiolucent jaw lesions that could be considered at differential diagnosis.

## 1. Introduction

The differential diagnosis of radiolucent lesions of the jaws can be a complicated act, as clinicians need to first distinguish whether the radiolucency is attributable to a normal anatomical structure, artefacts, or indeed pathology [1]. With the aid of artificial intelligence, this act could be initially done by means of computer algorithms and double-checked by qualified clinicians [2,3,4,5], as radiologists were among the groups of healthcare professionals with the highest prevalence of burnout [6]. Once it is confirmed as pathological, it can be broadly divided into congenital, developmental, and acquired conditions. The acquired conditions can be further categorized into a localized infection, spreading infection, trauma, cyst, tumor or tumor-like lesion, and bone-related lesion [7]. The cysts can be classified as odontogenic or non-odontogenic; tumors and tumor-like lesions can be odontogenic (benign or malignant), non-odontogenic intrinsic primary bone tumors, extrinsic primary tumors involving bone, secondary metastatic bone tumors, and lymphoreticular tumors of bone; and bone-related lesions can be giant cell lesions, osseous dysplasia (fibro-osseous lesions, FOL), and other lesions [8].

According to a retrospective study of 9723 radiolucent jaw lesions reported in Minnesota, the United States, the most common lesions were periapical granuloma (40.4%) and radicular cyst (33.1%) [9]. These are periapical inflammatory diseases with a pulpal origin, caused by a bacterial infection from necrotized dental pulp that passed through the root apex to involve the periradicular region in the alveolar bone. Odontogenic keratocysts (OKC; previously known as keratocystic odontogenic tumor, KCOT) accounted for 8.8% of the lesions, whereas other lesions accounted for around 2% or less each [9]. In another study with 4983 specimens with radiolucent jaw lesions, periapical inflammatory diseases similarly accounted for 72.8% of all lesions [10]. Clinicians need to be aware of the common lesions and appreciate the fact that their differential diagnoses can involve less common but clinically and radiologically similar entities. For example, a unilocular round radiolucency can represent a radicular cyst, but it can also represent an OKC or unicystic ameloblastoma, and only the histologic findings can clarify this [11]. Similarly, ossifying fibroma and fibrous dysplasia have similar radiological and even histological features but different patterns of disease progression [12]. It would be reasonable to anticipate that radiographically resembling lesions should be frequently mentioned together in the literature. Consequently, one should be able to reveal their relatedness by analyzing the semantic content of the literature. This has never been done before by prior bibliometric studies. The results should inform clinicians as to potential differential diagnoses, although readers should be aware that a final diagnosis can only be provided with a detailed clinical differentiation by a systematic analysis of clinical and radiographic features, blood chemistry, symptoms, and biopsy.

Therefore, the aim of the present study was to evaluate the dental literature reporting radiolucent jaw lesions. The objective was to reveal which radiolucent jaw lesions were frequently mentioned together in the reports and hence could be reasonably considered as differential diagnoses of the concerned lesions.

## 2. Materials and Methods

On 15 November 2021, the Web of Science Core Collection electronic database was queried with the following search string: TS = (radiolucent*) AND WC = (Dentistry Oral Surgery Medicine). This string searched for papers mentioning the word radiolucency and its derivatives in their title, abstract, or keywords. The papers were limited to those published in dental journals as classified by the database. A total of 1897 papers were retrieved and imported into VOSviewer (Leiden University, Leiden, the Netherlands), a bibliometric software, for the analyses.

First, the words in the titles and abstracts were analyzed. Those appearing in at least 10 papers were included. After producing an initial term map with default parameters in VOSviewer, the terms were screened. Generic terms and terms that were not diagnoses of radiolucent jaw lesions were manually removed. Ambiguous terms were merged (e.g., keratocystic odontogenic tumo(u)r was merged into odontogenic keratocyst). The final term map was then synthesized to visualize the relatedness of the diagnoses in terms of the frequency of co-occurrence, as indicated by the proximity between the terms as well as the thickness of the lines connecting them. Meanwhile, circle size and color represent publication count and citations per publication (CPP), respectively. Then, the above analytic procedures were repeated for author keywords. Keywords appearing in at least 3 papers were analyzed accordingly.

Additional literature searches were performed to explore potentially useful keywords that might be useful in differentiating frequently co-occurring lesions from the titles and abstracts (those co-occurring at least 4 times). Ameloblastoma and osseous dysplasia were taken as examples. Subsequently, the additional searches focused on the broader literature without confining themselves to papers mentioning the term “radiolucent”. For ameloblastoma, three searches were performed, respectively with (1) TS = (ameloblastoma* OR “odontogenic keratocyst*” OR “keratocystic odontogenic tum*”); (2) TS = (ameloblastoma* OR “dentigerous cyst*”); and (3) TS = (ameloblastoma* OR “radicular cyst*”). For osseous dysplasia, four searches were performed, respectively with (1) TS = (“osseous dysplasia*” OR osteomyelitis); (2) TS = (“osseous dysplasia*” OR “ossifying fibroma*”); (3) TS = (“osseous dysplasia*” OR odontoma*); and (4) TS = (“osseous dysplasia*” OR “fibrous dysplasia*”). Unfortunately, no prominent recurring clinical features or symptoms could be identified, as many of them might be mentioned in the full text which could not be analyzed by VOSviewer (elaborated as a limitation in the Discussion). Instead, relevant features or parameters would be extracted from resultant publications.

Since OKC was officially called KCOT by the World Health Organization during 2005–2017 [13], an exploratory analysis was conducted to reveal the number of papers within the entire literature that used OKC exclusively, KCOT exclusively, and both terms interchangeably in their title, abstract, or author keywords.

This study involves no animal or human subjects, so ethical approval was not needed.

## 3. Results

The top five most recurring journals for the 1897 papers were Oral Surgery, Oral Medicine, Oral Pathology and Oral Radiology (*n* = 212, citations per paper [CPP] = 22.0), Journal of Endodontics (*n* = 185, CPP = 28.2), Dentomaxillofacial Radiology (*n* = 100, CPP = 14.8), International Endodontic Journal (*n* = 96, CPP = 38.0), and Journal of Oral and Maxillofacial Surgery (*n* = 80, CPP = 14.4). The two other dental radiology journals, Oral Radiology (*n* = 25, CPP = 2.7) and Imaging Science in Dentistry (*n* = 23, CPP = 4.0), ranked 17th and 19^th^, respectively. In terms of countries, the top five most recurring ones were United States (*n* = 406, CPP = 23.6), Brazil (*n* = 243, CPP = 11.5), Japan (*n* = 146, CPP = 10.7), India (*n* = 133, CPP = 7.5), and China (*n* = 91, CPP = 19.9).

**Figure 1:**
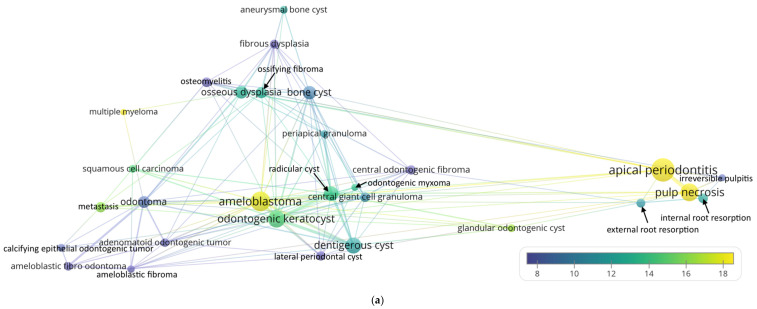

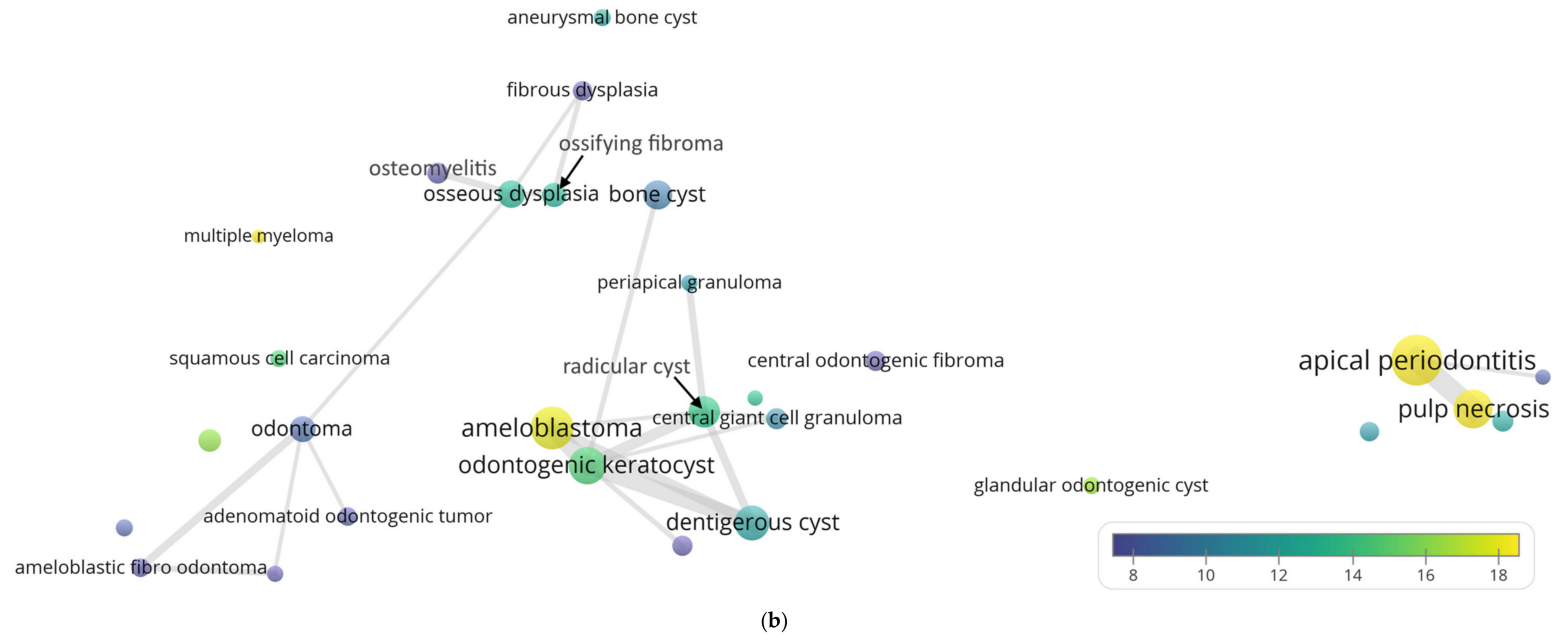
(**a**) Recurring radiolucent lesions mentioned in the title and abstract of the analyzed papers. (**b**) A simplified illustration that only connects lesions that co-occurred at least 4 times (the size variation of the lines was enhanced for clarity).

The recurring radiolucent lesions mentioned in the title and abstract of the papers are shown in Figure 1. The five most recurring radiolucent jaw lesions were apical periodontitis (*n* = 127, CPP = 31.4), ameloblastoma (*n* = 91, CPP = 17.5), odontogenic keratocyst (*n* = 69, CPP = 14.5), dentigerous cyst (*n* = 61, CPP = 11.7), and radicular cyst (*n* = 48, CPP = 13.7). The first three lesions were also among those with the highest CPP besides multiple myeloma (*n* = 10, CPP = 22.2) and glandular odontogenic cyst (*n* = 15, CPP = 16.1). In Figure 1a, odontogenic tumors are concentrated on the left; fibro-osseous lesions and bone cysts on the top; ameloblastoma and cystic lesions at the bottom; and periapical lesions with a pulpal origin on the right. To better highlight the frequent co-occurrences, the grey lines in Figure 1b only connect lesions that co-occurred at least four times together. Take ameloblastoma as an example. It was mentioned together with odontogenic keratocyst in 17 papers, with dentigerous cyst in 10 papers, and with radicular cyst in four papers. This implies that these are the most possible differential diagnoses for ameloblastoma from a bibliometric aspect. Take osseous dysplasia as another example. It was co-mentioned with osteomyelitis in seven papers, ossifying fibroma in five papers, odontoma in four papers, and fibrous dysplasia in four papers. This implies that these lesions should be considered when making a differential diagnosis.

**Figure 2:**
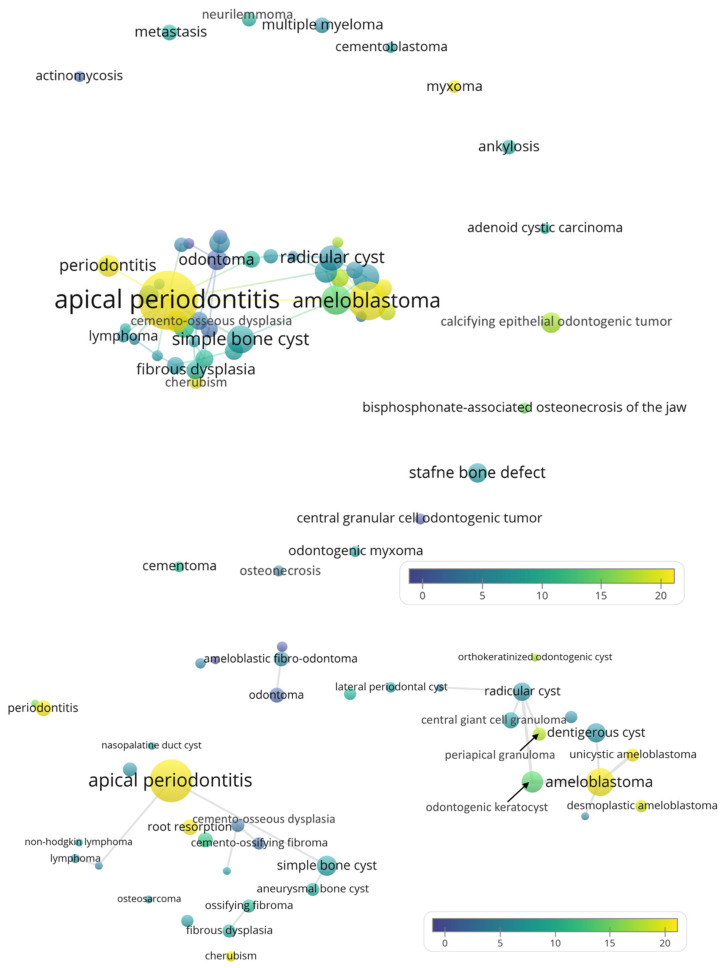
Recurring radiolucent lesions mentioned in the author keywords of the analyzed papers. Upper panel: the overall term map. Lower panel: zoomed-in view that focuses on the center of the map (the lines only connect lesions that co-occurred at least 2 times).

Some of the radiolucent lesions mentioned in the author keywords did not co-occur with others and were hence positioned at the periphery, as shown in Figure 2. Again, apical periodontitis (*n* = 82, CPP = 25.6) and ameloblastoma (*n* = 35, CPP = 19.5) were the foci with a high publication count and CPP. Notable lesions with high CPP included cherubism (*n* = 5, CPP = 19.6) and myxoma (*n* = 4, CPP = 19.8). Similar to the term map for the title and abstract, ameloblastoma was in close proximity to odontogenic keratocyst, radicular cyst, and dentigerous cyst, suggesting their radiographic resemblance. In terms of keywords, very few lesions co-occurred twice or more (Figure 2, lower panel), so this was not as illustrative as data from titles and abstracts.

Unfortunately, no relevant recurring clinical features or symptoms could be identified from the title, abstract, and keyword fields of the analyzed publications. To supplement this, relevant features or parameters were manually extracted from analyzed publications and listed in Table 1 for readers’ reference.

**Figure 3:**
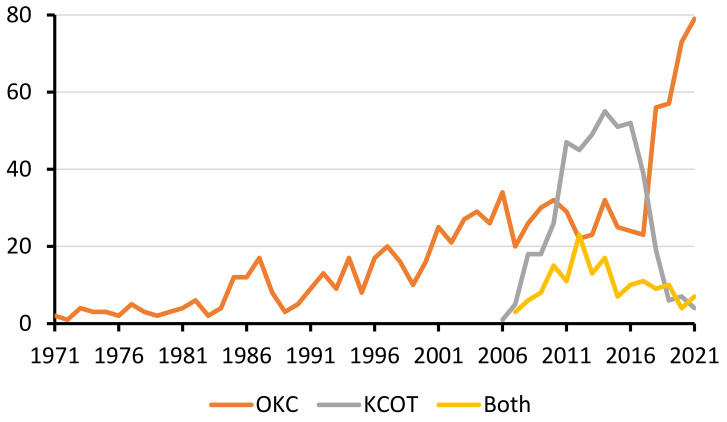
The use of the terms OKC, KCOT, and both in the entire literature.

Regarding the use of the terms OKC and KCOT in the entire literature, it was found that the exclusive use of the term OKC could be traced back to the early 1970s (Figure 3). The exclusive use of the term KCOT began in 2006 and declined sharply after 2017, consistent with the period of 2005–2017 when World Health Organization (WHO) called it KCOT. Among the 17 papers exclusively using the term KCOT published since 2019, India accounted for nearly half (*n* = 7). Three of them were published in surgery journals, and only one was in a dental journal.

## 4. Discussion

For the first time, this study demonstrated the applicability of co-word analysis for revealing a potential differential diagnosis of jaw lesions. It empowered junior clinicians and researchers to narrow down the list of lesions to be further considered when a radiolucent jaw lesion was encountered. There were two key points from the results. First, multiple lesions were identified from the title, abstract, and keywords, implying that relevant information was stored in these fields. Second, the frequently co-mentioned lesions, as illustrated in the Results section with ameloblastoma and osseous dysplasia, indeed offered radiologically reasonable differential diagnoses. For the first cluster, ameloblastoma was frequently mentioned together with OKC, dentigerous cyst, and radicular cyst. All of these entities are radiolucent lesions with well-defined borders, together with simple bone cyst, eosinophilic granuloma, and giant cell granuloma [26]. Due to their radiographic resemblance, recent studies developed deep learning object detection algorithms for their precise classification (ameloblastoma, OKC, dentigerous cyst, radicular cyst, simple bone cyst) [27,28,29]. Detailed clinical and radiographic examinations would provide qualitative factors that would help clinicians reach a reasoned differential diagnosis. For instance, both ameloblastoma and OKC may form a large lesion that spans across multiple teeth, but the former frequently causes root resorption of the teeth in proximity whereas the latter does not [30]. Meanwhile, a dentigerous cyst originates in and only surrounds the crown of an unerupted tooth, whereas a radicular cyst surrounds the root tip of an erupted tooth with an infected pulp. For the second cluster, osseous dysplasia was co-mentioned with osteomyelitis, ossifying fibroma, odontoma, fibrous dysplasia, and apical periodontitis. These entities usually possess a radiolucent rim or periphery, together with osteoid osteoma, osteoblastoma, and cementoblastoma [31]. Radiographically, it is particularly challenging to distinguish between osseous dysplasia and ossifying fibroma. However, osseous dysplasia is more commonly in close proximity with tooth apices or previous tooth extraction sites when compared to ossifying fibroma, but less frequently possesses a well-defined border [22]. Microscopically, the former often contains a cavernous-like vascularity associated with bone trabeculae and frequent hemorrhage, whereas the latter usually shows more cells arranged in a storiform pattern [23]. Meanwhile, it should be noted that apical periodontitis, being a highly recurring term from the analyzed literature set, is a consequence of endodontic infection that results in the local inflammation and widening of the periodontal ligamental space [32]. It is more confined to the periradicular region of a non-vital tooth compared to the more widespread osteomyelitis, osseous dysplasia, and ossifying fibroma. It is more common than other lesions, and hence quite reasonably had a higher publication count and CPP.

This study was new when compared to previous bibliometric studies that analyzed the semantic content of the literature, as the latter usually utilized the co-word analysis to cluster terms to identify the recurring research themes. For instance, it was reported that the neuroscience literature was broadly divided into two clusters, namely cellular, molecular or genetic neuroscience versus brain imaging [33]. Another example was an analysis of the antioxidant literature that concluded that the research focus shifted from vitamins and minerals to phytochemicals such as curcumin and resveratrol [34].

Of course, readers should be aware that the differential diagnoses obtained with the co-word analysis might not be exhaustive or very comprehensive. Indeed, radiolucent jaw lesions should be clinically differentiated with a systematic approach. The decision begins by checking if there are multiple discrete radiolucencies, along with checking the symmetry, margin definition, location, presence of malignant features, blood chemistry, and inflammation [35]. For the complete decision trees, readers may refer to [35]. Very rare lesions could be seldom mentioned by the literature, and hence they might be absent from the dataset. Even if they were included in the dataset, they might not be co-mentioned with other lesions, as illustrated in Figure 2. Meanwhile, disambiguation was a necessary procedure before conducting the bibliometric analysis. An analysis of authorship with initialized first names could pose a problem, as multiple authors might share identical initials, especially for Chinese names [36]. In this study, different names referring to the same lesion were combined. For example, OKC was officially called KCOT by the WHO during 2005–2017 [13]. Therefore, the mentions of OKC and KCOT were merged, and both American and British spellings of “tumo(u)r” were merged. In this study, it was found that some Indian papers continued to exclusively use the term KCOT after it had been renamed OKC in 2017. This implied that the nomenclature of lesions might have a geographical difference, similar to the nomenclature of anatomical terms such as mandibular canal (versus inferior alveolar canal, etc.) [37]. The renaming was due to clinico-histopathological considerations, unlike certain gene names being renamed due to paronomasia [38] or to avoid autocorrection errors by software such as Excel [39]. Meanwhile, it was pleasing to observe the sharp decline of the term KCOT, implying that in this case the healthcare academia largely and swiftly followed the latest changes in the nomenclature. This could have been difficult, considering that the terminology could be ingrained in researchers’ knowledge base, resurfacing from time to time as it was embedded in medical web search engines [40].

The current study had several limitations. The analyzed literature was relatively small. In theory, the entire dental literature could be included for the analysis. However, that would involve many papers not dealing with radiolucent jaw lesions, and many more recurring terms would be manually screened and excluded, rendering this unfeasible for the author. On the other hand, the Web of Science database only stored information from the title, abstract, and keywords; and, similarly, VOSviewer was only capable of analyzing semantic content from these fields. This meant that it was not possible to extract information from the full text of the identified papers, which might mention more lesions as well as the clinical features and symptoms of the main related different morbidities (e.g., in the introduction and discussion parts of the full text). Meanwhile, to achieve the most accurate results that cover the majority of the relevant literature, the literature search must be performed in a systematic way.

In conclusion, the co-word analysis on the radiolucent jaw lesion literature was demonstrated to reveal the common and reasonable differential diagnoses of the concerned lesions, which may be helpful for clinicians and researchers when they make clinical and research decisions.

## 5. Conclusions

The co-word analysis, a form of bibliometric analysis, could demonstrate a relatedness of radiolucent jaw lesions that could be considered at differential diagnosis.

## Figures and Tables

**Table 1:** Relevant features or parameters that might differentiate or distinguish radiographically resembling lesions.

Features or Parameters	Reference
**Ameloblastoma as compared to OKC**	
Bone expansion in buccolingual dimension and absence of high-density areas on CT	[14]
Early dental epithelial markers were differentially overexpressed in ameloblastoma, whereas squamous epithelial differentiation markers were the most differentially overexpressed genes in OKC	[15]
Higher ADC level in diffusion-weighted magnetic resonance imaging	[16]
**Ameloblastoma as compared to dentigerous cyst**	
RANK-positive and RANKL-positive cells	[17]
Ameloblastoma as compared to dentigerous cyst and OKC	
Higher number of RANK-positive than OPG-positive cells. Opposite is true for the latter two pathologies.	[17]
Positive calretinin staining	[18,19]
**Ameloblastoma as compared to radicular cyst and OKC**	
Higher ADC level in diffusion-weighted magnetic resonance imaging	[20]
**Osseous dysplasia as compared to osteomyelitis**	
Presence of fibroblastic stroma with bone and cementum-like structures	[21]
**Osseous dysplasia as compared to ossifying fibroma**	
More commonly in close proximity with tooth apices or previous tooth extraction sites	[22]
Less frequently possesses a well-defined border	[22]
The former often contains cavernous-like vascularity associated with bone trabeculae and frequent hemorrhage, whereas the latter usually shows more cells arranged in a storiform pattern	[23]
**Osseous dysplasia as compared to odontoma**	
Occurring in older patients (over 30 years of age) and related to the root	[24]
Osseous dysplasia as compared to fibrous dysplasia and ossifying fibroma	
The former one is usually symptomless, whereas the latter two present painless swelling	[25]
The former one contains woven bone, whereas the latter two contain a mixture of woven bone and cementum-like materials	[25]

ADC, apparent diffusion coefficient. CT, computed tomography. OPG, osteoprotegerin. RANK, receptor activator of nuclear factor κB. RANKL, RANK-Ligand.

## Data Availability

Data is available from the Web of Science platform.
